# Monopolar Radiofrequency Ablation of Thyroid Nodules: A Prospective Austrian Single-Center Study

**DOI:** 10.1089/thy.2017.0547

**Published:** 2018-04-01

**Authors:** Harald Dobnig, Karin Amrein

**Affiliations:** ^1^ Thyroid Endocrinology Osteoporosis Institute Dobnig/Schilddrüsen Endokrinologie Osteoporose Institut Dobnig, Graz, Austria.; ^2^ Department of Internal Medicine, Division of Endocrinology and Diabetology, Medical University of Graz, Graz, Austria.

**Keywords:** RFA, thyroid nodule, thermal ablation, cystic nodule, toxic nodule, nodule shrinkage

## Abstract

***Background:*** Monopolar radiofrequency ablation is currently deemed an exotic treatment option for benign thyroid nodules in many central European countries. The aim of this study was to evaluate prospectively the safety and efficacy of this method in a large patient cohort following its introduction in Austria.

***Methods:*** Peri- and post-interventional complications were analyzed for 277 patients. Efficacy was determined for 300 and 154 nodules at 3 and 12 months post treatment, respectively. All treatments were performed with an internally cooled 18G radiofrequency electrode using a free-hand, “moving-shot” technique following subcutaneous and local perithyroidal anesthesia.

***Results:*** Mean patient age (*SD*) was 52 ± 12.9 years (75% female), and overall mean baseline nodule volume (*SD*) was 13.8 ± 15.9 mL. Nodules were visible in 62.8% of patients, 40% had a symptom score ≥4 on a 10-point visual analogue scale, and 14.4% had hyperthyroidism. Mean overall nodule volume reduction rates (VRR) at 3 and 12 months were 68 ± 16% and 82 ± 13%, respectively (*p* < 0.001). At 12 months, 81% of nodules exhibited a VRR of ≥70%, with 10%, 6%, and 2% of nodules showing VRRs of 60–70%, 50–60%, and ≤50%, respectively. Subgroup analysis according to baseline nodule size (≤10 mL to >30 mL) or baseline nodule composition (solid, mixed, cystic) revealed significantly higher VRRs for smaller and cystic nodules. Moreover, nodule shrinkage was accompanied by significantly improved symptom and cosmetic scores after 3 and 12 months (*p* < 0.001). Of 32 hyperthyroid patients, 27 (84%) were euthyroid, four had subclinical hyperthyroidism, and one had subclinical hypothyroidism at last follow-up. Post-procedural complications were absent in 83% of patients, minimal in 12.9%, moderate and reversible in 3.2% (1.8% voice change, 0.7% hyperthyroidism, 0.3% wound infection treated with antibiotics, 0.3% epifascial hematoma), and irreversible in 0.7% (one case with hypothyroidism and one with a wound infection treated by surgery).

***Conclusions:*** It is concluded that a single treatment course with monopolar radiofrequency ablation is both safe and highly effective in terms of nodule volume reduction, relief of local symptoms, and (in patients with hyperthyroidism) restoration of euthyroid function. In no case was a prescription of thyroid medication required among those patients who were euthyroid at baseline.

## Introduction

The vast majority of thyroid nodules present no clinical problem, as 90% do not increase in size over time (1). On the other hand, because of the high prevalence of thyroid nodules, the 10% that do grow can lead to functional or cosmetic symptoms and warrant further examination and treatment in a significant number of individuals. The most effective first treatment choice for symptomatic benign thyroid nodules is surgery. Outpatient treatment alternatives that avoid surgery and conserve normal thyroid function have, however, recently become available. These so-called thermoablative methods induce local thermodestruction, leading to nodule shrinkage and improvement of local symptoms. Monopolar radiofrequency ablation (RFA) is presently the best-documented thermoablative method. Its availability, however, remains restricted, offered mainly by a few research groups worldwide (largely located in South Korea, Italy, and China) that since 2006 have reported at least 18 interventional studies on the effects of a single treatment course with a monopolar, internally cooled system (2–19). Other studies addressing complications and limitations of RFA complete the picture of this systematically evolving method over time (20). To foster general acceptance of this new method, prospective studies in larger and more differentiated cohorts (including those from other geographical and cultural regions) are needed, in particular because a number of the available studies are limited by retrospective design (4,6,9–12,14,17), small sample size (2–7,9,10,13–15,19), or small average volume of treated nodules (2,4,12). Moreover, little is known concerning the efficacy of a single, monopolar RFA treatment course for toxic nodules (19).

The purpose of the present study was to investigate prospectively the effects of a single RFA treatment in Austria where this method has not yet been studied. Moreover, the study was designed to be large enough in scope to enable a representative prospective analysis of peri- and post-interventional complications, as well as a subgroup analysis based on initial nodule size, composition and thyroid function.

## Methods

This prospective analysis was approved by the local Ethical Committee of the Medical University of Graz and formed part of a diploma thesis by Katja Kaiblinger (EK Number 29-084 ex 16/17). The patients signed a written seven-page information and consent form and also agreed to the analysis and publication of the obtained data.

All patients undergoing RFA at the authors' institute (Schilddrüsen Endokrinologie Osteoporose Institut Dobnig) from April 2014 to June 2017 were included. All except three were Caucasian.

Only benign nodules (i.e., Bethesda class II on fine-needle aspiration cytology or fine-needle capillary cytology) based on two separate aspirates under ultrasound guidance were treated. A core needle biopsy was performed for 3% of patients because of a “not representative” cytology report. Clearly toxic nodules identified by thyroid scans were not subjected to fine-needle aspiration.

All dominant nodules caused either functional symptoms or psychological disturbances and/or were of cosmetic concern, were growing significantly over the last year, or were hyperfunctioning. The great majority of patients presented with an external report recommending thyroid surgery (*N* = 214; 77.2%) and were seeking an alternative treatment option. Most of the patients were self-referrals (85.4%), with some referred by internists or general practitioners (7.1%) and others by thyroid specialists (7.5%). Patients were seeking alternatives to thyroid surgery for a number of reasons (see Results section). Exclusion criteria included ill-defined nodule margins and far distal locations, which prevented ablation of the entire nodule. RFA was also declined for patients with multinodular goiters for whom, despite an anticipated significant volume reduction following RFA treatment, an overall unsatisfactory outcome in terms of improvement of local symptoms or thyroid function was predictable or likely. Further exclusion criteria were pregnancy, the presence of a cardiac pacemaker, or a history of neck or trunk external beam radiation.

### Clinical evaluation

As suggested in a previous consensus statement (21), symptoms (using a 10-point visual analog scale [VAS]) and cosmetic concerns were routinely classified as part of the pre-procedural work-up. To obtain a “symptom score,” patients were asked the following question: “To what overall degree does the nodule bother you cosmetically or in terms of functional impairment or reduced psychological well-being on a scale of 0 to 10?” An objective “cosmetic score” was obtained using the following scale: 0, no palpable mass; 1, a palpable mass with no cosmetic problem; 2, a visible nodule during neck extension and/or during swallowing; 3; readily visible thyroid nodule.

### Biochemical evaluation

Laboratory tests prior to RFA comprised calcitonin measurement (<10 ng/dL), a complete blood count, blood coagulation tests, as well as thyrotropin (TSH; reference range 0.4–4.0 mIU/L), free triiodothyronine (fT3; 2–8 pmol/L), and free thyroxine (fT4; 9–28 pmol/L) with standard thyroid laboratory work repeated at 3 and 12 months post RFA.

### RFA intervention

All patients underwent a single RFA session in an outpatient setting in a room specially dedicated to ultrasound-guided interventional treatments. Ultrasound was performed using a 4–15 MHz linear matrix array probe (ML6-15-D) on a Logiq-S8 (GE Healthcare, Wauwatosa, WI). Nodule and thyroid lobe volume were calculated with the following equation: *V* (mL) = length × width × depth × 0.525. Nodule volume reduction rate was calculated using the following equation: VRR (%) = [(baseline volume – final volume) × 100]/baseline volume. Care was taken during follow-up ultrasound-based nodule volume measurements to place the distance calipers at the anatomical position used for baseline measurements. The solid component of each thyroid nodule was evaluated as a percentage of tens. Nodules were categorized as “solid or predominantly solid” (solid component ≥70%), “mixed” (solid component 40–60%), or “cystic or predominantly cystic” (solid component ≤30%) based on their appearance upon ultrasound examination. Baseline nodule sizes were categorized arbitrarily into four size groups: ≤10 mL (“small”), >10 to 20 m: (“medium”), >20 to 30 mL (“large”), and >30 mL (“very large”).

Patients were given a 400 mg dexibuprofen tablet 30 minutes prior to RFA. They were then placed on an operation bed in the supine position with hyperextended neck and with knees resting on leg elevator wedges to provide a comfortable positioning throughout the intervention. A grounding pad was applied to each ventral thigh. At no time were patients given intravenous solutions or drugs. Patients were routinely monitored for noninvasive blood pressure, EKG, SpO2, pulse rate, and respiration (VISMO; Nihon-Kohden, Tokyo, Japan) by an intensive care nurse who was also responsible for generator power adjustments during RFA and proper functioning of the cooling system. A second registered nurse assisted the RFA procedure. All RFA interventions were recorded for documentation purposes.

RFA was administered using internally cooled 18G electrodes (STARmed, Seoul, Korea), 7–10 cm in length, and with a 7–15 mm active tip size according to nodule size, composition, and function, powered by the VIVA RF generator (480 KHz; STARmed). A small local subcutaneous 2% xylocaine depot was created ventral to the nodule, followed by careful pericapsular infiltration (in both cases with a 23G needle). In patients who presented with thyroid nodules in contact with or in close proximity to the trachea, the spatium surrounding the trachea was also infiltrated. Vascular structures along the approach route of the electrode were carefully inspected. A “transisthmic” route was routinely used, except for two patients with prominent veins located anteromedial to the thyroid capsule for whom a “lateral” approach of the electrode was chosen. Larger nodules (usually ≥4 cm in length) required two insertion points. Nodules with a liquid cystic component were aspirated under transisthmic ultrasound guidance using a 21G needle prior to RFA intervention, while those with a viscous content were drained beforehand using a 16G or 14G needle and sometimes a vacuum extractor. RFA itself was performed using the “moving-shot” technique developed by Baek *et al*. (3,22). Initial power output was, in each case, set at 40 W and, subject to formation of sufficient hyperechoic microbubbles and active tip size, raised in 20 W increments until appropriate results were obtained. RFA was then continued until all parts of the nodule had been thoroughly treated and color Doppler examination confirmed an absence of measurable blood flow. The elapsed time needed for RFA intervention starting with local anesthetic injection was recorded at this point, together with the “generator time” and the energy administered to the nodule (in kcal). A flexible cold pack was applied with moderate force to the treated side of the neck immediately after treatment. The RFA intervention was concluded with another recorded ultrasound examination to confirm the absence of hematomas or other apparent abnormalities. Following another five minutes of cold-pack compression the patient was then observed for half an hour, during which time he/she continued cold-pack treatment and was given a 25 mg prednisolone tablet. Moreover, patients were provided with 300 mg dexibuprofen tablets (one for the evening of the intervention day, and three to be taken as needed the following day) and two 25 mg prednisolone tablets (for the morning of two consecutive days) as a preventative measure to minimize local swelling. All patients with a history of thyroid surgery or large nodules had a pre-procedural laryngoscopic examination, while those with voice changes following RFA were monitored by laryngoscopy until normal vocal cord function was confirmed. Routine follow-up visits, including ultrasonography and standard thyroid function tests (TSH, fT4, fT3), were scheduled for 3 and 12 months post RFA. Hyperthyroid patients were asked to stop antithyroid medication on the day of ablation and to report thyroid function tests results back to us in a month. A thyroid scan was moreover performed on these patients at month 3 to look for any remaining hyperfunctional nodule remnants.

### Statistical analysis

Statistical analysis was performed using IBM SPSS Statistics for Windows v24.0 (IBM Corp., Armonk, NY). Descriptive analyses (mean, median, standard deviation, range) were computed on clinical variables and thyroid- and nodule-related parameters. For changes of within-group data (baseline vs. 3 or 12 months), parameters were analyzed using the Wilcoxon signed-rank test. To compare group mean values, a *t*-test was used in cases in which a Shapiro–Wilk test indicated normal distribution after log transformation of the parameters. Equivalent nonparametric tests were otherwise used (Mann–Whitney *U*-test, Kruskal–Wallis test). Spearman's rank correlation coefficient was used to evaluate the level of correlation between initial nodule volume and total energy administered. Analysis of variance was used to calculate time-related differences in the effects of VRR since the introduction of this method. Differences were considered statistically significant at *p*-values of ≤0.05.

## Results

### Clinical characteristics and nodule volume

Patient characteristics and clinical and relevant nodule-related data are presented in Table 1. The overall mean nodule volume of approximately 13.8 mL corresponds to a spherical nodule diameter of 3.0 cm. Table 2 provides an overview of objective nodule-related findings (i.e., size and growth), as well as symptom-related findings and the arguments most often cited by patients as having led them to seek an alternative to thyroid surgery.

**Table 1.** Patients and Nodule Characteristics

Number of patients	277
Age	52 (12.9)
Sex, female/male (%)	215/62 (77.6%/22.4%)
Number of nodules (total)	361
Number of patients with documented recommendation for thyroid surgery	214 (77.2%)
Number of patients with number of treated nodules	
1	201 (72.5%)
2	53 (19.2%)
≥3	23 (8.3%)
Patients with toxic nodules	55 (19.8%)
Patients with hyperthyroidism (with or without thiamazole/propylthiouracil)	40 (14.4%)
Mean nodule volume (mL)	13.8 (15.9)
Mean largest nodule diameter (cm)	3.4 (1.3)
Mean total thyroid volume (mL)	32.6 (20.9)
Mean volume of thyroid lobe (side of lesion; mL)	23.8 (17.6)
Mean volume of thyroid lobe (contralateral to side of lesion; mL)	8.5 (5.6)
Patients with bilateral RFA	46 (16.6)

RFA, radiofrequency ablation.

**Table 2.** Clinical Background and Patients' Personal Reasons for Seeking RFA Treatment (*N* = 277)

*Relevant clinical background*	*Number of patients*	*%*
Documented external recommendation for thyroid surgery	214	77.2
Symptom score ≥4 (VAS)	110	40
Nodule visible with neck in normal position	122	44
Nodule visible with neck extended	51	18.6
Significant recent growth of nodule	80	28.8
Hyperthyroid patients with toxic nodules	40	14.4
*Most frequently expressed personal reasons*		
“I don't want thyroid surgery to be performed” (unrelated to certain fears)	141	51
“I have normal thyroid function and do not want to take a medication”	136	49
Doctor recommended total thyroidectomy	33	11.9
History of thyroid surgery	17	6.1
Fear of surgery and/or general anesthesia	15	5.4
Fear of voice change	15	5.4
Negative experience with thyroid surgery by a family member or by a friend	13	4.7
Relevant comorbidities	13	4.7
Cannot afford to stay away from home for longer at the moment	13	4.7
Recommendation to “avoid lifting” post surgery is not realistic at the moment	12	4.3
Feeling of being too old or too young for thyroid surgery	7	2.5

VAS, visual analog scale.

**FIG. 1.**
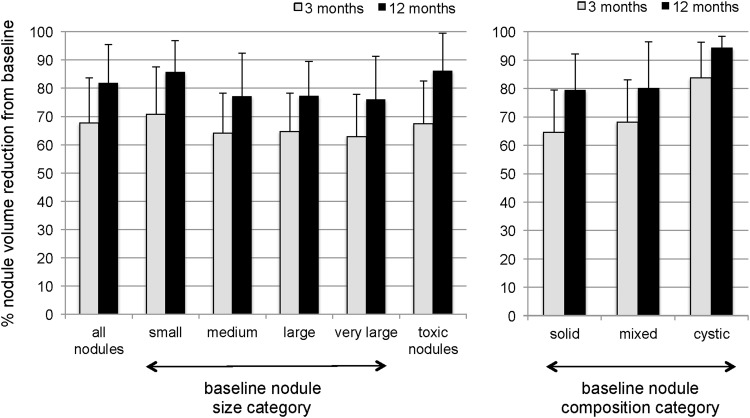
Subgroup analysis of nodule volume reduction ratio at 3 and 12 months according to different baseline nodule characteristics based on initial size and nodule composition (mean ± *SD*).

Most nodules were either solid or predominantly solid (74.4%). The remainder were mixed (12.1%) or cystic/predominantly cystic (13.5%). The mean volume of solid or predominantly solid nodules (*N* = 267) was 13.6 ± 15.9 mL (median = 7.8 mL; range 1–109 mL), the mean volume of mixed nodules (*N* = 43) was 14.1 ± 15.0 mL (median = 10.1 mL; range 1–84 mL), and the mean volume of cystic or predominantly cystic nodules (*N* = 48) was 15.9 ± 17.1 mL (median = 8 mL; range 2–83 mL). Data for nodule subgroup analysis are based on initial size and composition (Table 3 and Fig. 1). The VRRs for smaller and cystic/predominantly cystic nodules were on average 8.8% and 14.5% higher than for the other groups 12 months post RFA (*p* < 0.001).

**Table 3.** Subgroup Analysis of Nodule Volume Reduction of Different Nodule Size and Nodule Composition Categories at Baseline

	*Baseline size of treated nodules*				
	*Nontoxic nodules*				
	*Small*	*Medium*	*Large*	*Very large*	*Toxic nodules*
Nodule volume (mL)	≤10 mL	>10 to ≤20 mL	>20 to ≤30 mL	>30 mL	
Baseline	4.6 ± 2.8 (178)	13.6 ± 2.4 (62)	24.5 ± 2.5 (28)	47.7 ± 17.8 (46)	8.7 ± 7.0 (47)
3 months	1.4 ± 1.3 (147)^***^	4.8 ± 2.2 (53)^***^	8.5 ± 3.4 (24)^***^	17.7 ± 12.7 (37)^***^	2.9 ± 1.6 (39)^***^
12 months^a^	0.7 ± 0.8 (72)^***^	2.9 ± 2.0 (28)^***^	5.3 ± 3.0 (17)^***^	12.3 ± 15.2 (22)^***^	2.1 ± 4.7 (15)^***^
Nodule volume compared to baseline (%)					
3 months^b^	–70.8 ± 16.7 (147)^***^	–64.1 ± 14.2 (53)^***^	–64.7 ± 13.5 (24)^***^	–62.9 ± 14.9 (37)^***^	–67.4 ± 15.2 (39)^***^
12 months^c^	–85.7 ± 11.1 (72)^***^	–77.2 ± 15.2 (28)^***^	–77.3 ± 12.2 (17)^***^	–76.0 ± 15.3 (22)^***^	–86.1 ± 13.4 (15)^***^

	*Baseline composition of nontoxic nodules*		
	*Solid or predominantly solid*	*Mixed*	*Cystic or predominantly cystic*
Solid part of nodule (%)	(>70 to 100)	(>30 to 70)	(0 to 30)
Nodule volume (mL)			
Baseline	14.1 ± 16.5 (232)	15.8 ± 16.7 (34)	15.9 ± 17.1 (48)
3 months	5.5 ± 7.9 (194)^***^	6.0 ± 8.2 (28)^***^	2.8 ± 3.6 (39)^***^
12 months^a^	3.9 ± 7.7 (102)^***^	4.1 ± 8.4 (19)^***^	1.0 ± 1.2 (18)^***^
Nodule volume compared to baseline (%)			
3 months^d^	–64.6 ± 14.8 (192)^***^	–68.1 ± 14.9 (27)^***^	–83.7 ± 12.6 (39)^***^
12 months^d^	–79.4 ± 12.8 (102)^***^	–80.2 ± 16.3 (19)^***^	–94.3 ± 4.1 (18)^***^

Data shown are mean (*SD*), with number of patients in parentheses.

^a^ All 12-month values were significantly different from respective 3-month measurements (*p* < 0.001).

^b^ Compared to small nodules, *p*-values were significantly different from: medium nodules (*p* = 0.01), large nodules (*p* = 0.05), and very large nodules (*p* = 0.005).

^c^ Compared to small nodules, *p*-values were significantly different from: medium nodules (*p* = 0.05), large nodules (*p* = 0.01), and very large nodules (*p* = 0.005).

^d^ Compared to cystic/predominantly cystic nodules, volume reductions for solid/predominantly solid and mixed nodules were different (*p* = 0.001 at 3 and 12 months).

^***^ *p* < 0.001 versus respective baseline values.

### Technical aspects

Initial nodule size (mL) correlated positively with energy delivered (kcal) to the nodule: *R* = 0.78 (*p* = 0.001). Average RFA treatment duration was 39.8 ± 15.3 minutes, the mean generator time was 7.4 ± 5.1 minutes, and the mean Wattage was 56 ± 19 per nodule. Seven and 10 mm active tips were used most frequently (42% and 38% of cases, respectively). Five and 15 mm active tips were used in 13% and 7% of cases, respectively.

**FIG. 2.**
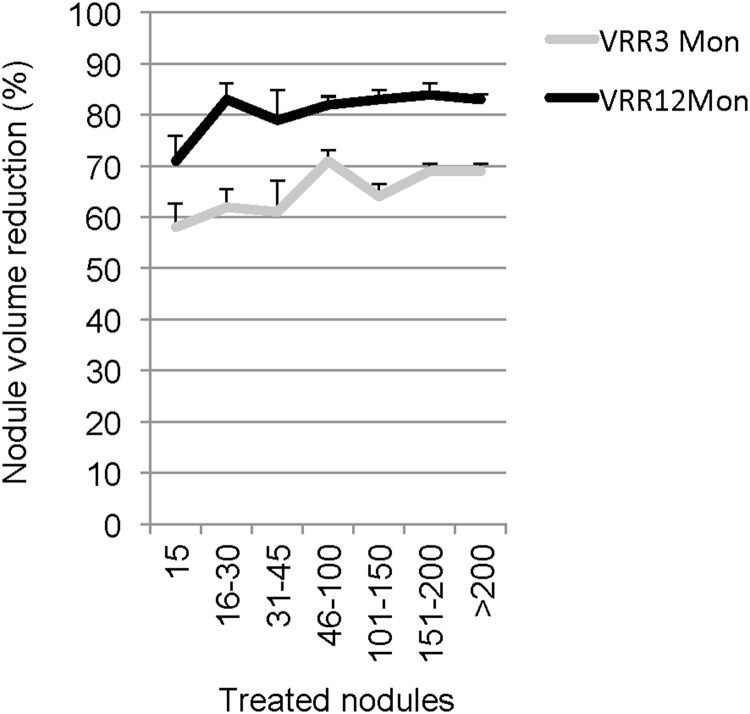
The graph shows nodule VRR for consecutively treated nodules at 3 and 12 months since introduction of the RFA method. Analysis of variance was significant for VRR at three months (*p* = 0.01) but not at 12 months (*p* = 0.08). The trend over time suggests a VRR difference of approximately 10% and a learning curve for monopolar RFA that reached a plateau quite early.

Nodule VRR is the most important indicator of ablation success. Based on this parameter, the study calculated whether there were differences in averaged VRRs within certain time intervals after the introduction of RFA. These results suggested that the plateau of the learning curve was reached quite quickly. The difference in VRR between early and later ablation success was roughly 10%, which was significant by analysis of variance (ANOVA) of results at three months (*p* = 0.01), though not significant by ANOVA at 12 months (*p* = 0.08; Fig. 2).

**FIG. 3.**
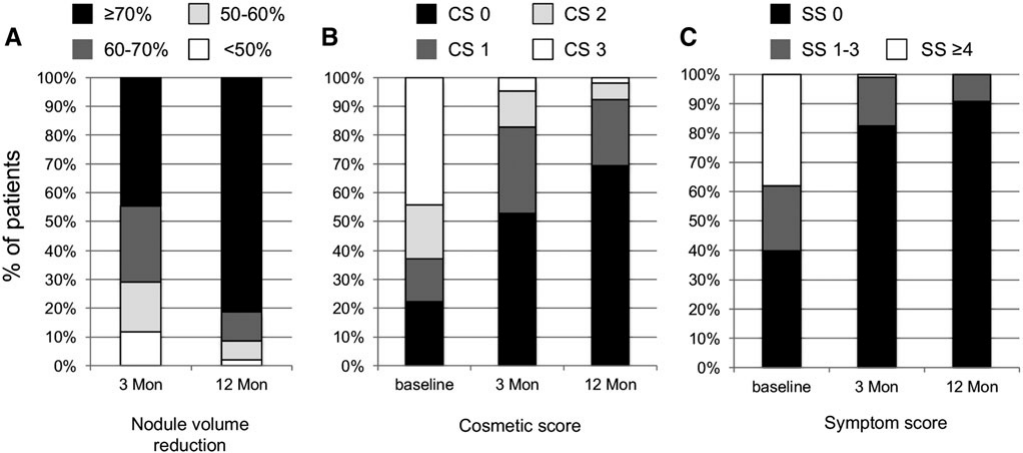
Percentage of patients and their outcomes in terms of volume reduction ratio (VRR) and cosmetic and symptom scores at baseline and at 3 and 12 months. Cosmetic score: CS 0, no palpable nodule; CS 1, nodule palpable; CS 2, nodule visible during swallowing and/or neck extension; CS 3, nodule easily visible. Symptom score was measured using a visual analog scale (0–10). Nodule volume reduction categories were significantly different between 3 and 12 months. Cosmetic and symptom scores at 3 and 12 months were significantly different from baseline scores, as well as from each other (*p* < 0.001).

An overview of ablation success for the entire study population at 3 and 12 months is provided in Figure 3. At 12 months post RFA, >80% of the patients had a nodule VRR of ≥70%.

### Cosmetic score and symptom score

Nodule shrinkage was also accompanied by an improvement in cosmetic and symptom scores. Prior to RFA, 62.8% of the patients had a nodule that was visible with or without neck extension. At three months post RFA, this percentage decreased to 17.1%, and at 12 months, it decreased to 7.1% (*p* < 0.001). The difference between 3 and 12 months was also significant (*p* < 0.001). Patients with a symptom score of ≥3 (52.9%) or ≥4 (38.2%) at baseline also reported improvements at month 3 (5.2% and 1% with symptom score ≥3 or ≥4) and at month 12 (2.8% and 0%) after RFA. This difference between the scores at 3 and 12 months was also significant (*p* < 0.001).

### Hyperthyroid patients

**FIG. 4.**
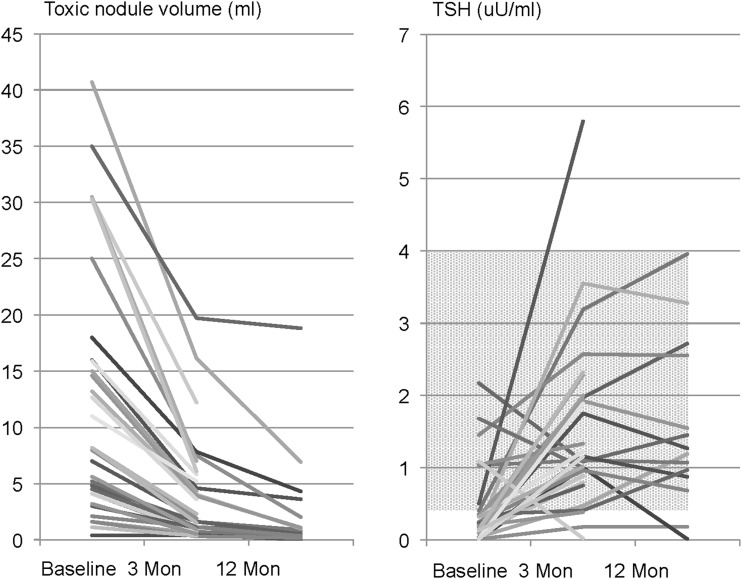
Individual results of hyperthyroid patients at baseline and development of volume of toxic nodules and TSH values over time. Approximately half of patients had antithyroid medication at baseline, none at follow-up. Of 32 patients with follow-up measurements, 27 (84.3%) were euthyroid at last visit, one (3.1%) patient developed subclinical hypothyroidism, and four had subclinical hyperthyroidism (all had manifest hyperthyroidism at baseline).

Fifty-five patients had autonomous nodules on thyroid scans at baseline, of whom 15 had a euthyroid TSH value. The remaining 40 patients had subclinical or overt hyperthyroidism, with a total of 47 toxic nodules. Among these patients, 17 patients were taking antithyroid medication at the time of RFA. Toxic nodules were solid (77%) or of mixed composition (23%). The energy delivered to toxic nodules (kcal/mL) was significantly higher compared to nontoxic nodules (0.56 ± 0.35 vs. 0.35 ± 0.28 kcal/mL; *p* < 0.001). Table 3 shows the development of mean toxic nodule volume over time. Figure 4 provides individual absolute toxic nodule volumes, as well as TSH values over the course of the study. Follow-up data are available for 32 patients (18 at month 3 and 14 at month 12). Of these, 27 (84.3%) became euthyroid, one (3.1%) developed subclinical hypothyroidism, and four (12.5%) had subclinical hyperthyroidism at their last visit. The patient with subclinical hypothyroidism initially had a decompensated toxic nodule and showed a VRR of 85% and a rise in TSH level from 0.5 mIU/L (with thiamazole) to 5.8 mIU/L (without thiamazole). Total thyroid volume was rather small (7.2 mL), and thyroid antibodies were absent. Thyroxine replacement therapy has not yet been started. Of the four patients with subclinical hyperthyroidism after RFA, three had pronounced overt hyperthyroidism with large toxic nodules before RFA and exhibited VRRs of 60%, 76%, and 92%, accompanied by a marked improvement in technetium uptake in thyroid scans. These three patients were subsequently successfully managed with a low-dose oral ^131^I treatment (5 mCi or 185 MBq) and finally had normal TSH values without need of any thyroid-specific medication. In patient 4, the baseline toxic nodule no longer showed increased uptake in the thyroid scan on follow-up, and the TSH increased from 0.19 to 0.38 mIU/L. The final functional outcome of this patient, however, remains open at present.

### Peri- and post-procedural complications

In 89.9% of cases, a single local perithyroidal infiltration of local anesthetic was sufficient to allow for ablation with only minimal discomfort and no noteworthy pain (see Tables 4 and 5). In the remaining cases, RFA was interrupted and infiltration repeated, usually at a different topographical site (i.e., peritracheal). RFA was completed as scheduled in all cases, except for one. In this case, an intramuscular blood vessel was injured by probe insertion. Ablation was completed at a second visit.

**Table 4.** Peri-Procedural Complications (*N* = 277)

	*Patient number*	*%*
Pain		
Grade 0	249	89.9
Grade 1	22	7.9
Grade 2	6	2.2
Hematoma (intramuscular)	4	1.4
Hypotension	2	0.7
Post-interventional diarrhea	1	0.3

Pain grade 0, local anesthesia was given once, and there was only little discomfort; pain grade 1, local anesthesia had to be repeated once; pain grade 2, local anesthesia had to be repeated two to three times.

**Table 5.** Post-Procedural Complications (*N* = 277)

*Severity*	*Degree of ailment*	*%*	*Complication*	*Patient number*	*%*	*Time to recovery (days)*
Grade 0	None	83.0		230	83.0	
Grade 1	Minimal (reversible)	12.9				
			Hematoma (subcutaneous)	12	4.3	7–21
			Dysphagia	9	3.2	1–7
			Neck stiffness	4	1.4	1–30
			Elevated temperature	4	1.4	7–14
			Tiredness	2	0.7	2–3
			Pain	2	0.7	2
			Palpations	1	0.3	3
			Neck swelling	1	0.3	17
			Headaches	1	0.3	3
Grade 2	Moderate (reversible)	3.2				
			Voice change (temporary)	5	1.8	3–90
			Hyperthyroidism	2	0.7	30–310
			Wound infection (antibiotics)	1	0.3	21
			Hematoma (epifascial)	1	0.3	14
Grade 3	Irreversible	0.7				
			Hypothyroidism	1	0.3	None
			Wound infection (surgery)	1	0.3	None

A detailed list of the post-procedural complications is provided in Table 5. Graves' disease developed in one patient five months after RFA, and thyroid function did not return to normal until 10 months later. A second patient exhibited subclinical hyperthyroidism for four weeks but was euthyroid at the three-month visit. All voice changes completely resolved after three days (one patient), one month (two patients), and two-and-a-half months (two patients). An epifascial hematoma developed three weeks after ablation and reached 5 mm in diameter but disappeared spontaneously. A wound infection acquired by a breastfeeding mother was successfully treated with antibiotics. A second wound infection was acquired by a forest worker who, contrary to our advice, worked in the woods the day after RFA and was later obliged to undergo thyroid surgery because of the wound infection. One female patient with hyperthyroidism at baseline developed a TSH of 5.8 mIU/L after a VRR of her toxic nodule of 85%. She is presently symptom free and therefore under treatment-free observation.

### TSH, fT3, and fT4 measurements

The euthyroid patient group exhibited a small but significant rise in serum TSH levels from 1.42 ± 0.77 mIU/L at baseline to 1.82 ± 1.08 mIU/L at three months post RFA (*N* = 182; *p* < 0.001) and 1.80 ± 1.01 mIU/L at 12 months (*N* = 96; *p* < 0.001). Mean fT4 levels remained between 15.0 pmol/L (baseline) and 15.8 pmol/L, and mean fT3 levels between 4.77 pmol/L (baseline) and 4.83 pmol/L during follow-up. Neither of these changes is considered clinically relevant.

The TSH level for patients with hyperthyroidism was 0.43 ± 0.56 mIU/L at baseline (with 17 patients taking antithyroid medications), 1.50 ± 1.13 mIU/L at three months, and 1.55 ± 1.15 mIU/L at 12 months (all without antithyroid medication; all *p*-values vs. baseline <0.001). fT4 levels showed a small non-significant decrease at 12 months: 16.0 ± 2.8 vs. 16.3 ± 5.5 pmol/L at baseline (*p* = 0.08). fT3 changes were more pronounced, with decreases from the baseline of 5.74 ± 2.15 pmol/L to 4.80 ± 0.49 pmol/L at three months (*p* < 0.001) and 4.65 ± 0.79 pmol/L at 12 months (*p* < 0.05).

## Discussion

This study is an analysis evaluating the effects of a single monopolar RFA treatment of benign thyroid nodules in one of the largest prospectively reported cohorts to date. The observed overall efficacy of nodule volume reduction of 68% after three months and 82% after 12 months compares favorably to previous smaller studies (18) and is of the same effect size as the largest study to date (12). The latter, however, was of retrospective nature and recruited patients with on average markedly smaller nodules (mean volume 5.4 mL). Limited group size and restricted inclusion criteria have to date, with exceptions (2,15), precluded a more detailed analysis of effect size based on baseline nodule characteristics such as size and composition. The present results demonstrate a higher VRR for “smaller” nodules than “large” nodules 12 months after RFA (roughly 10% higher) and that “cystic/predominantly cystic” nodules exhibit a significantly better treatment effect compared to “solid” and “mixed” nodules (a difference in VRR of approximately 15% after one year). The relatively large overall effect size of around 80% volume reduction in all investigated subgroups nevertheless argues against the use of morphological criteria based on these two characteristics (size and composition) as important selection criteria for ideal RFA candidates.

The most important outcomes, however, are the absolute decrease in the percentage of patients with significant symptoms (VAS ≥4) from 38.2% to 0%, and the reduction in the number of “visible” nodules from 62.8% to 7.5% one year post RFA. It is not surprising that practically all other studies have also reported improvements in symptoms and cosmetic scores, since they also reported marked volume effects by RFA (18,23). However, a weak correlation is often observed between nodule size and the degree of symptoms, that is, patients with easily visible nodules frequently experience almost no symptoms, while others with nodules a few milliliters in size complain of overt symptoms. In the authors' view, this phenomenon, which is likely dependent on neck anatomy, nodule topography and composition, pain threshold, cultural attributes, as well as other factors (24), should be considered in data analyses. Therefore, such data are presented broken down into “categories” of a symptom and a cosmetic score rather than as an “averaged number” for the entire group.

A recently published analysis summarizing the effects of RFA on autonomous thyroid nodules found a normalization of thyroid function at last follow-up in 24–82% of cases (19). Two of the six studies, however, used a multi-tined expandable probe (25,26), and in three of them RFA was performed more than once (26–28). The reasons underlying the 84% success rate achieved in the present study can only be speculated, but it is believed that a smaller average baseline volume of toxic nodules, as well as delivery of a significantly higher amount of energy per milliliter nodule, are the most likely reasons. Euthyroid function was not achieved in four patients with large and partially cystic toxic nodules. The VRR following RFA in these patients was, however, sufficiently high to enable successful treatment of these nodules with a small dose of radioiodine (5 mCi ^131^I). This combined treatment modality thus restored normal thyroid function without the need for surgery or subsequent thyroid replacement therapy.

RFA was well tolerated with pericapsular and, in some cases, accompanying peritracheal anesthetic infiltration. While most groups use a “local anesthesia at the puncture site,” others perform additional conscious sedation with 2–5 mg of midazolam or even general anesthesia (14,16,17,19). In the authors' view, there is little justification for the latter, since pericapsular infiltration provides excellent analgesia, allows frequent reassessment of vagal and recurrent laryngeal nerve function, and eases patient discharge following the post-interventional observation period. Many patients are quite anxious prior to RFA, which can also be stressful for the operator and the team. To counteract this, it is important to provide a calm atmosphere and to seek conversation, especially during positioning and preparation for RFA. At the authors' institution, patient anxiety has been lowered by the assistant holding one of the patient's hands throughout the entire ablation procedure while monitoring the patient.

With 3.2% of patients showing moderate and reversible and 0.7% irreversible sequels, the overall incidence of complications following RFA seems acceptable. Complications encountered in a large, multicenter retrospective analysis from South Korea also occurred at low frequency, but were notably slightly different in nature (21). For instance, no wound infections or patients with hyperthyroidism were reported, and the incidence of temporary voice changes was also slightly lower than in the present study (1.02% vs. 1.8%). Other reported incidence rates of voice changes following monopolar RFA range from 0.5% to 4.7% (20). On the other hand, no episode of vomiting, skin burns, or brachial plexus injury was observed in the current study. The overall findings are consistent with a recent systematic review based on 3409 patients, which also concluded that RFA is a safe and well-tolerated treatment modality for benign thyroid nodules (20).

Aside from several strengths such as the prospective study design and cohort size, the present study also has some noteworthy limitations. Because all ablations were performed by a single operator (H.D.), the results do not account for variations in experience and skill, which is certainly a factor in most interventional studies. Moreover, this study exhibits a weakness shared by many studies investigating other thermoablative methods, namely a lack of longer-term follow-up data (i.e., exceeding one year). Knowledge regarding possible recurrence rates of both cold and toxic nodule growth is currently scarce or even absent (23,29), but is crucial for estimating long-term cost–benefit ratios.

In summary, the results of the present study are consistent with the existing literature and confirm the safety of RFA in general and the efficacy of a single treatment course of monopolar RFA in a large central European cohort. The results also provide some potentially significant data for future discussions regarding possible selection criteria for patients with thyroid nodules that could be treated with RFA.
